# Functional Impacts of the BRCA1-mTORC2 Interaction in Breast Cancer

**DOI:** 10.3390/ijms20235876

**Published:** 2019-11-23

**Authors:** Kimiko L. Krieger, Wen-Feng Hu, Tyler Ripperger, Nicholas T. Woods

**Affiliations:** 1 Eppley Institute for Research in Cancer and Allied Diseases, Fred & Pamela Buffett Cancer Center, University of Nebraska Medical Center, Omaha, NE 68198, USA; kimiko.krieger@unmc.edu (K.L.K.); wenfeng.hu@unmc.edu (W.-F.H.); ripperger@email.arizona.edu (T.R.); 2 Department of Emergency Medicine, College of Medicine, University of Nebraska Medical Center, Omaha, NE 68198, USA; 3 Department of Immunobiology, College of Medicine, University of Arizona, Tucson, AZ 85724, USA

**Keywords:** BRCA1, mTORC2 complex, BRCT domain, DNA damage response, protein-protein interactions, RICTOR, SIN1, PRR5

## Abstract

Deleterious mutations in Breast Cancer 1 (*BRCA1*) are associated with an increased risk of breast and ovarian cancer. Mutations in the tandem BRCA1 C-terminal (tBRCT) protein domain disrupt critical protein interactions required for the faithful repair of DNA through homologous recombination, which contributes to oncogenesis. Our studies have identified RICTOR, PRR5, and SIN1 subunits of the mammalian target of rapamycin complex 2 (mTORC2) as interacting partners with the tBRCT domain of BRCA1 leading to the disruption of the mTORC2 complex. However, the interplay between mTORC2 signaling and BRCA1 function in the DNA damage response (DDR) remains to be determined. In this study, we used protein interaction assays to determine the binary interactions between the tBRCT domain and mTORC2 subunits, evaluated the impact of mTOR inhibition on the transcriptional function of the tBRCT, evaluated the impact of mTOR signaling on BRCA1 recruitment to DNA damage-induced foci and determined the breast cancer cell line response to mTOR inhibition dependent upon *BRCA1* expression and mutation. This study determined that PRR5, RICTOR, and SIN1 could each independently interact with the BRCA1 tBRCT. Inhibition of mTORC1, but not mTORC1/2, increases BRCA1 transcriptional activation activity. Treatment with pan-mTOR inhibitor PP242 diminishes DNA damage-induced γH2AX and BRCA1 foci formation. Breast cancer cells lacking expression of functional BRCA1 are more sensitive to mTOR inhibitors. These data suggest that mTOR signaling is required for BRCA1 response to DNA damage and breast cancer cells lacking BRCA1 are more sensitive to pan-mTOR inhibition. This work suggests chemotherapeutic strategies using mTOR inhibitors could be tailored for patients that lack functional BRCA1.

## 1. Introduction

Breast cancer is the most frequently diagnosed cancer in women with an estimated 266,120 new cases diagnosed and approximately 40,920 fatalities in 2018, per National Cancer Institute’s Surveillance, Epidemiology, and End Results (SEER) Program. In breast cancer, roughly 5–10% of cases are attributed to inherited mutations found in the tumor suppressor *BRCA1* [1,2,3], and inactivation of the wild type allele promotes tumor initiation [4]. Inherited mutations of *BRCA1* increases the lifetime risk of developing breast cancer from 11% in the general population to 50–80% and ovarian cancer from 1.4–2.5% to 15–60% [3]. Impaired expression of *BRCA1* can also occur through epigenetic silencing, leading to an increased risk of breast cancer [5,6].

Mutational analysis of *BRCA1* led to the discovery of the modular BRCT domain and its characterization as an essential component of the tumor suppressive function of BRCA1 [7]. Since its discovery, the BRCT domain has been found in only 23 human proteins, most of which have been functionally annotated to participate in DNA damage response and repair [8,9,10,11,12,13,14,15]. Functional and structural characterization of the BRCA1 tBRCT has revealed that it is essential for the recognition of DNA damage-induced serine phosphorylations by binding the consensus sequence phospho-SXXF (pSXXF) [16,17]. BRCA1, as well as other BRCT domain-containing proteins, have also been noted to have phosphorylation-independent interactions with its targets [18,19,20]. The BRCA1 tBRCT domain acts as a scaffold enabling recruitment of interacting proteins to sites of DNA damage [21,22,23]. While the tBRCT domain has no intrinsic enzymatic activity, it is essential for organization of macromolecular complexes that mediate the DDR [24,25,26]. As a scaffolding domain, the function of the tBRCT can be characterized by its protein interactions. Our previous work has sought to define the tBRCT interactome, including the BRCA1 tBRCT, using yeast two-hybrid, tandem affinity purification coupled to mass spectrometry (TAP-MS), and literature curation [19]. Delineation of the protein-protein interactions mediated by the tBRCTs is essential to understanding the network of protein interactions contributing to the regulation of the DDR through distinct molecular pathways, which has the potential to identify novel therapeutic strategies to treat or prevent cancer.

The previous TAP-MS data published by our lab identified three members of the mTORC2 complex (RICTOR, PRR5, and SIN1) that interacted with the BRCA1 tBRCT domain [19]. Of the seven tBRCT domains from different proteins that were interrogated (BARD1, BRCA1, ECT2, LIG4, MDC1, PAXIP1, TP53BP1), only the tBRCT domain from BRCA1 was found to interact with the mTORC2 complex proteins. The mTORC2 complex activates the pro-survival kinase Akt by directly phosphorylating Ser473, thereby promoting its kinase activity [27]. We previously discovered that BRCA1 tBRCT prevents Ser473 phosphorylation by dissociating the members of the mTORC2 complex from the mTOR kinase. This contributes to hyperactivation of the Akt pathway observed in breast cancer cells lacking BRCA1 expression [19]. The mTORC2 complex is involved in many other processes of the cell, such as growth, proliferation, survival, cytoskeletal organization, apoptosis, metabolism, and stress response [27]. However, the impact of mTORC2 signaling on the function of BRCA1 and how this impacts the DDR has not been evaluated.

The PI3K/AKT/mTOR pathway is hyperactivated in more than 70% of breast tumors, but therapeutic targeting can produce unexpected results due to the complex nature of its regulation [28]. Therefore, biomarkers are required to reliably target this pathway in cancer patients. Given the role of BRCA1 in the regulation of mTORC2, the mutation and expression status of BRCA1 may provide a biomarker. In addition, mTORC1 signaling inhibition by rapamycin suppresses double-strand break repair [29], targets of mTOR show decreased phosphorylation upon inhibition of ATM [30], and mTORC2 protects yeast from replication-associated DNA damage [31]. These findings clearly implicate mTORC1/2 in the DNA damage response network, yet the interplay between BRCA1 and mTORC2 signaling remains poorly defined. Since loss of *BRCA1* leads to the hyperactivation of mTORC2, it may be possible that breast cancer cells lacking *BRCA1* could be dependent upon mTORC2 signaling and more sensitive to its inhibition. Hence, our goal for this study was to test the relationship between BRCA1 status and sensitivity to mTORC2 inhibition in breast cancer.

Currently, there has not been an mTORC2 specific inhibitor developed. For this study, we used a small panel of mTOR inhibitors, including rapamycin, PP242, and PKI-179. Rapamycin is an mTOR inhibitor that targets the FATC domain of mTOR [32]. This inhibitor successfully targets mTORC1. PP242 is a second-generation mTOR inhibitor that specifically targets the mTOR kinase, thus inhibiting both mTORC1 and mTORC2 [33]. PKI-179 is a dual pan-PI3K, mTOR inhibitor, which inhibits both mTOR complexes as well as PI3K [34,35].

In the present study, we sought to evaluate the impact of mTORC2 signaling on the DNA damage response through BRCA1. We determined BRCA1 tBRCT directly interacted with RICTOR, PRR5, and SIN1. The transcriptional activation of BRCA1 was enhanced following mTORC1 inhibition, but not mTORC2. Furthermore, pan-mTOR inhibition led to defects in the recruitment of BRCA1 to DNA damage foci. In addition, breast cancer cells lacking BRCA1 expression were more sensitive to mTOR inhibition. These results suggest stratification of breast cancer treatment utilizing mTOR inhibitors based on BRCA1 status may improve their efficacy. 

## 2. Results

### 2.1. The BRCA1 tBRCT Domain Directly Interacts with Each RICTOR, PRR5, and SIN1 mTORC2 Complex Subunits

**Figure 1:**
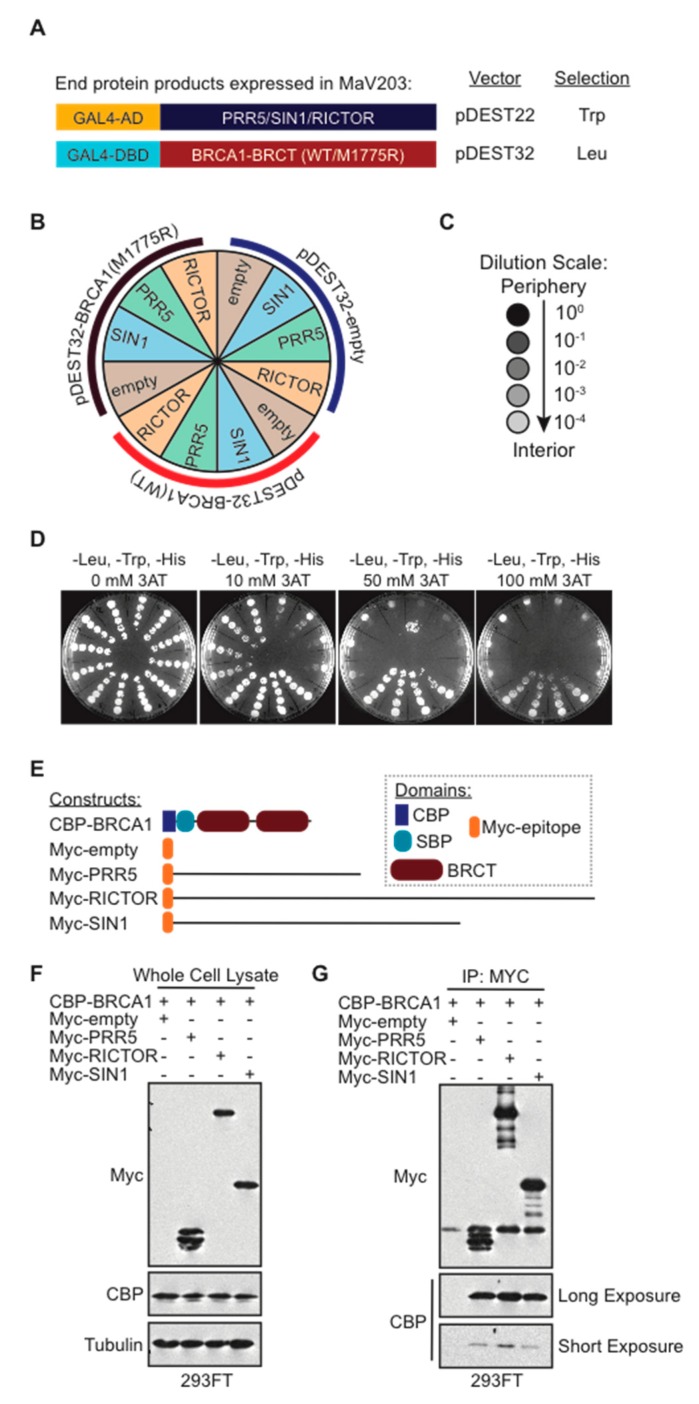
Functional BRCA1 BRCT domain specifically interacts with each of the members of the mTORC2 complex. (**A**) Representation of the N-terminus GAL4-DBD and GAL4-AD protein fusion constructs (cloned in Gateway cloning vectors pDEST32 and pDEST22, respectively) used for the Y2H experiment. pDEST32 constructs have a tryptophan selection marker, and pDEST22 constructs have a leucine selection marker. Competent MaV203 yeast cells were co-transformed with the indicated gene pairs using the subcloning scale protocol; (**B**) experimental plate schematic of the Y2H interaction study displaying an empty vector, BRCA1 WT, and BRCA1 mutant M1775R; (**C**) dilution scale for yeast cultures; (**D**) plates of yeast co-transfected with pDEST22 constructs of members of the mTORC2 complex (PRR5, SIN1, and RICTOR) and pDEST32 constructs (empty vector, BRCA1 WT, and BRCA1-M1775R). Each master colony was grown in selective -His, -Leu, and -Trp liquid medium to saturation with increasing concentrations of 3-amino-1,2,4-triazole (3AT); (**E**) schematic of constructs used for co-immunoprecipitation experiment; (**F**) Western blot displaying whole cell lysates of CBP-BRCA1 and myc-PRR5, -SIN1, and -RICTOR constructs co-overexpressed in 293FT cells; (**G**) Co-immunoprecipitation of CBP-tagged BRCA1 and myc-tagged PRR5, RICTOR, and SIN1.

The experiments performed in this study were designed to identify the mTORC2 subunit(s) that directly interacted with the BRCA1 BRCT domain. The Yeast Two-Hybrid (Y2H) system can identify a binary interaction between two proteins. To decipher the binary protein interactions, we implemented the ProQuest Two-Hybrid system (Invitrogen). The BRCA1 tBRCT domain baits used in this assay included wild type (WT) or the negative control M1775R mutant that disrupted the phospho-peptide binding. These tBRCT bait constructs were cloned into the pDEST32 vector to generate GAL4 DNA binding domain (GAL4-DBD) fusions (Figure 1A). The bait proteins (PRR5, SIN1, and RICTOR) were cloned into the pDEST22 vector to generate GAL4 activation domain (GAL4-AD) fusions (Figure 1A). The survival and growth of the yeast colony on selective media (-Leu, -Trp, -His) are dictated by intermolecular interactions that induce gene expression. In these Y2H experiments, there were twelve different gene expression combinations tested (Figure 1B). The yeast strain MaV203 used in this study allowed the interrogation of three selection markers, of which we focused on HIS3, which is optimal for relatively weak interactions, commonly found for BRCT domain interactions. Liquid cultures were serially diluted ten-fold to test limiting numbers of cells in the samples and plated with increasing amounts of HIS3 inhibitor 3-amino-1,2,4-triazole (3AT) (Figure 1C,D). With increasing amounts of 3AT up to 100 mM, the interactions between BRCA1 WT and empty vector, PRR5, RICTOR, and SIN1 constructs remained. These results suggest that the interaction of BRCA1 with mTORC2 proteins was dependent on a functional tBRCT domain.

To confirm these interactions in a mammalian model, TAP-tagged BRCA1 tBRCT and Myc-tagged RICTOR, PRR5 and SIN1 constructs were co-transfected into human embryonic kidney 293FT cells and their expression was confirmed by immunoblotting (Figure 1E,F). Immunoprecipitation was performed using α-MYC antibody. The immunoprecipitation showed that each RICTOR, PRR5, and SIN1 construct could pull-down the TAP-tagged BRCA1 tBRCT domain. However, CBP blots developed at a shorter exposure time indicated that the interaction between the BRCA1-BRCT domain and Rictor may be slightly stronger than the interactions between PRR5 and SIN1 (Figure 1G). Together, these results suggest that RICTOR, PRR5, and SIN1 each interact with the BRCA1 tBRCT domain and that the interactions can occur independent of the other mTORC2 subunits.

### 2.2. Rapamycin Increases BRCA1 Transcriptional Activation Activity

**Figure 2:**
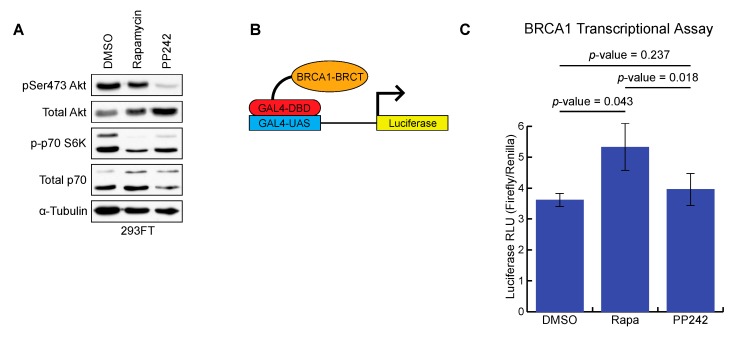
Rapamycin increases BRCA1 transcriptional activation activity. (**A**) Western blot displaying mTORC1 and mTORC2 activity via phosphorylation of Ser473 on Akt and Thr421/Ser424 phosphorylation of p70 S6K; (**B**) Schematic of BRCA1 transcriptional activation assay; (**C**) Bar graph representing the data generated from the BRCA1 transcription activation assay across three independent samples (DMSO control, rapamycin, and PP242) (*n* = 3 independent experiments). Data represented as the mean +/- standard error of the mean. A student’s t-test was used to determine the statistical significance.

BRCA1 is a transcriptional coactivator and is responsible for the transcription of a number of genes, including IFNγ response genes, NF-κB target genes, p21^WAF1/Cip1^, p27^Kip1^, and Gadd45α [36]. In addition, BRCA1 has also been found to repress ERα gene expression, which has been a point of focus for hormone-related cancers, such as breast cancer [36]. The tBRCT domain of BRCA1 is the mediator for the transcriptional activation activity of BRCA1 and this functionality can be used to classify variants of unknown significance [37,38]. The impact of rapamycin on mTORC1 inhibition and PP242 on mTORC1 and mTORC2 inhibition was confirmed by Western blot (Figure 2A). The inhibition of the mTORC1 and mTORC2 pathways was measured by p70 S6K phosphorylation and Akt phosphorylation at Ser473, respectively (Figure 2A). 

We utilized the established BRCA1 tBRCT transcriptional activation assay based on the BRCA1 (aa 1315–1863) N-terminal fusion with GAL4-DBD, which recruits the construct to the Gal4-upstream activation signal (GAL4-UAS) and promotes transcription of the luciferase gene when functional BRCA1 tBRCT is present (Figure 2B) [38]. Following transfection of the BRCA1 BRCT domain, cells were treated with either rapamycin (mTORC1 inhibitor), PP242 (pan-mTORC1/2 inhibitor), or DMSO control. Luciferase reporter activity was plotted onto a bar graph in a ratio of Firefly to control Renilla luciferase for each of the three experiments (Figure 2C). Rapamycin treatment significantly increased BRCA1 transcriptional activation activity compared to DMSO control. Treatment with pan-mTOR inhibitor PP242 did not affect BRCA1 transcriptional activation activity compared with a DMSO control. These results suggest that inhibition of mTORC1 with rapamycin treatment increases BRCA1 transcription activation, but this effect is abrogated when both mTORC1 and mTORC2 are both inhibited by PP242.

### 2.3. mTORC2 Activity Prevents Cisplatin-Induced Cell Death in MCF-10A Cells 

**Figure 3:**
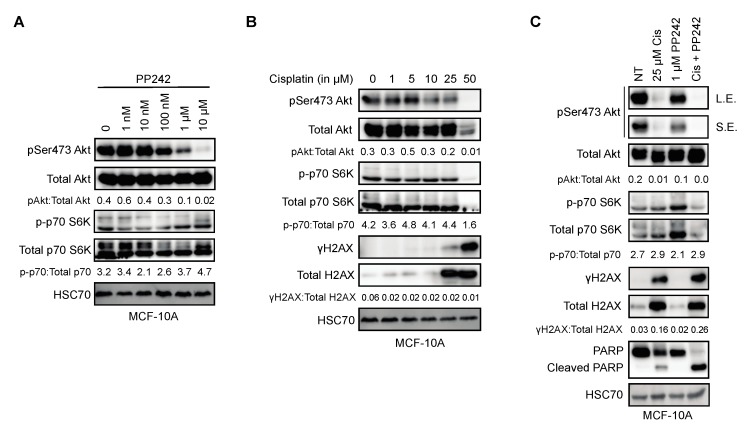
mTORC1/2 activity prevents Cisplatin-induced cell death in MCF-10A cells. (**A**) Western blot displaying effects on mTOR signaling during a dose escalation of PP242 treatment in MCF-10A cells; (**B**) Western blot displaying effects of mTOR signaling on a dose escalation of cisplatin treatment in MCF-10A cells; (**C**) Western blot displaying effects on mTOR signaling and cell death during non-treated, Cisplatin, PP242, and Cisplatin + PP242-treated MCF-10A cells.

PP242 was the preferred mTOR inhibitor for this study because of its marked specificity to the mTOR kinase as opposed to other second-generation mTOR inhibitors that have off-target effects on other PIKK family kinases, such as ATM, ATR, and DNA-PK [39]. To determine the concentration of PP242 that effectively inhibits Akt phosphorylation at Ser473, a dose-escalation experiment was conducted on MCF-10A cells (Figure 3A). Akt Ser473 phosphorylation was found to be reduced at 1 μM PP242 in MCF-10A cells (Figure 3A). In addition, we chose to use the DNA damaging agent Cisplatin to explore mTORC2 effects in response to DNA damage because it is known to preferentially induce cancer cell death when functional BRCA1 is absent [40]. A dose-escalation of Cisplatin was used to determine that a 25 μM concentration was required to generate a response to DNA damage, indicated by the appearance of phosphorylated H2AX (γH2AX) (Figure 3B). We also observed that MCF-10A cells exhibited a dose-dependent decrease of Akt Ser473 phosphorylation up to 25 μM Cisplatin and a nearly complete loss of Akt and p70 S6K expression at 50 μM Cisplatin where the DDR was the highest as evidenced by γH2AX expression levels (Figure 3B). To evaluate the impact of mTORC1/2 signaling on cell death in response to Cisplatin, we performed combination treatments using 25 μM Cisplatin, 1 μM PP242 and Western blotting for cleaved PARP, which was cleaved during the execution of apoptosis (Figure 3C). MCF-10A cells treated with both Cisplatin and PP242 had little to no Akt phosphorylation and a significant increase in PARP cleavage in comparison with Cisplatin alone (Figure 3C). This suggests that mTORC1/2 signaling promotes resistance to Cisplatin-induced cell death.

### 2.4. Repression of mTOR Signaling Significantly Reduces γH2AX-BRCA1 Foci Formation

**Figure 4:**
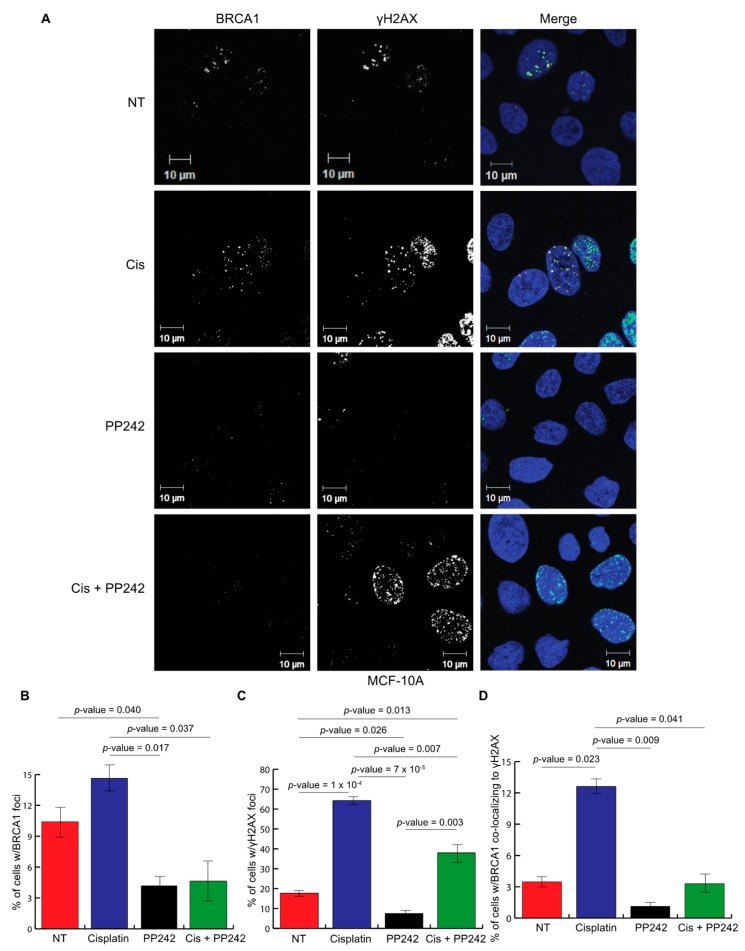
Repression of mTOR signaling significantly reduces γH2AX-BRCA1 foci formation. (**A**) Representative immunofluorescence of γH2AX, BRCA1 and DAPI (green, red, and blue, respectively, in a merged view) in MCF-10A cells with a control (DMSO), 50 μM Cisplatin, 1 μM PP242, 10 nM Rapamycin, Cisplatin with PP242, and Cisplatin with Rapamycin for 24 h; (**B**) graph representing the percentage of nuclei containing ≥5 BRCA1 foci with and without Cisplatin, PP242, and Cisplatin + PP242 treatment (*n* = 3 independent experiments, each with ≥ 300 cells evaluated/experiment). Data represented as the mean +/− standard error of the mean. Student’s t-test was used to determine the statistical significance; (**C**) graph representing the percentage of nuclei containing ≥5 γH2AX foci with and without Cisplatin, PP242, and Cisplatin + PP242 treatment (*n* = 3 independent experiments, each with ≥ 300 cells evaluated/experiment). Data represented as the mean +/− standard error of the mean. Student’s t-test was used to determine the statistical significance; (**D**) graph representing the percentage of nuclei containing ≥ 5 BRCA1-γH2AX foci with and without Cisplatin, PP242, and Cisplatin + PP242 treatment (*n* = 3 independent experiments, each with ≥300 cells evaluated/experiment). Data represented as the mean +/− standard error of the mean. Student’s t-test was used to determine the statistical significance.

It has been previously established that BRCA1 co-localizes with γH2AX to recruit DNA damage response proteins to repair DNA double strand breaks [41]. To evaluate how the inhibition of mTORC1/2 signaling may play a role in BRCA1 function and the DNA damage response, nonmalignant breast cell line MCF-10A was used to observe foci formation for both γH2AX and BRCA1. MCF-10A cells were treated with a control, Cisplatin (a DNA damage-inducing agent commonly used as chemotherapy for breast cancer), PP242, and Cisplatin in combination with PP242 (Figure 4A). Foci formation of both BRCA1 and γH2AX were analyzed as well as BRCA1 localization to γH2AX (Figure 4B–D). As expected, there was an induction of BRCA1 foci, γH2AX foci, and BRCA1-γH2AX co-localization in response to treatment with Cisplatin. However, there was a significant decrease in BRCA1 foci, γH2AX foci, and BRCA1-γH2AX co-localization in response to PP242 or the combination of Cisplatin plus PP242 in comparison to the Cisplatin-only treated cells. These results suggest that mTORC1 and mTORC2 signaling is required for DNA damage-induced γH2AX and BRCA1 foci formation.

### 2.5. BRCA1 Loss Sensitizes Breast Cancer Cells to mTOR Inhibition

**Figure 5:**
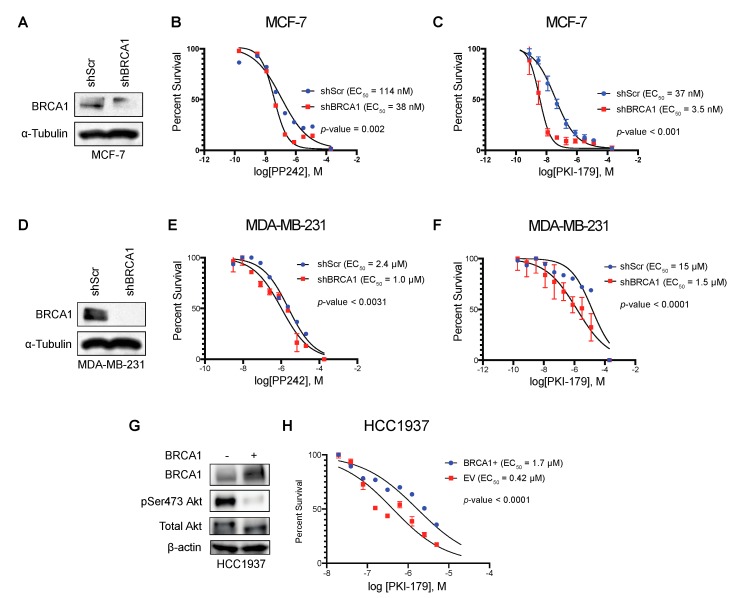
Breast cancer cells with BRCA1 loss are sensitive to mTOR inhibition. (**A**) Western blot displaying confirmation of BRCA1 knockdown in MCF-7 cells; (**B**) sigmoidal curve representing the percentage of survival from the cell viability assay used to determine the half maximal effective concentration (EC_50_) of PP242 in MCF-7 cells (*n* = 3 independent experiments); (**C**) sigmoidal curve representing the percentage of survival from the cell viability assay used to determine the half maximal effective concentration (EC_50_) of PKI-179 in MCF-7 cells (*n* = 3 independent experiments); (**D**) Western blot displaying confirmation of BRCA1 knockdown in MDA-MB-231 cells; (**E**) sigmoidal curve representing the percentage of survival from the cell viability assay used to determine the half maximal effective concentration (EC_50_) of PP242 in MDA-MB-231 cells (*n* = 3 independent experiments); (**F**) sigmoidal curve representing the percentage of survival from the cell viability assay used to determine the half maximal effective concentration (EC_50_) of PKI-179 in MDA-MB-231 cells (*n* = 3 independent experiments); (**G**) Western blot displaying confirmation of BRCA1 overexpression in HCC1937 cells; (**H**) Sigmoidal curve representing the percentage of survival from the cell viability assay used to determine the half maximal effective concentration (EC_50_) of PKI-179 in HCC1937 cells with and without full length BRCA1 (*n* = 3 independent experiments).

We have previously established that impaired BRCA1 expression is associated with the hyperactivation of Akt [19]. The Akt pathway provides a strong pro-survival signal that can impair the chemotherapeutic response in cancer. Therefore, we sought to examine the therapeutic effects of mTOR inhibition in relation to BRCA1 expression in breast cancer cell lines. MCF-7 breast cancer cells with BRCA1 knockdown were more sensitive to pan-mTOR inhibitor PP242 and pan-PI3K/pan-mTOR inhibitor PKI-179 than cells with the non-targeting scrambled control (Figure 5A–C). Similar results were also observed in the triple-negative breast cancer cell line MDA-MB-231 (Figure 5D–F). To further validate our findings, we used a genetic model comparing HCC1937, which naturally expresses non-functional, truncated BRCA1, and HCC1937(BRCA1+) cells with restored full-length BRCA1 expression. We treated this isogenic cell line pair with PKI-179 and found that HCC1937 cells were more sensitive to PKI-179 than the HCC1937 (BRCA1+) (Figure 5G–H). These data suggest that breast cancer cells that lack functional BRCA1 are more sensitive to mTORC1/2 inhibition.

## 3. Discussion

The crosstalk between mTORC2 and BRCA1 arising from their protein interaction is important to understand breast and ovarian cancer susceptibility and improve treatment options based on a patient’s *BRCA1* mutation status. The ability of the BRCA1 tBRCT domain to disrupt the mTORC2 complex is a direct indication of the coordination between the DNA damage response and mTORC2-Akt pro-survival signaling. The results in this study confirmed that the BRCA1 tBRCT domain interacts with mTORC2 complex subunits RICTOR, PRR5, and SIN1 individually, although the affinity of the RICTOR interaction may be slightly higher than PRR5 or SIN1. If the tBRCT domain can interact with RICTOR, PRR5 and SIN1 simultaneously, this would suggest a large interaction area on the tBRCT domain is being occupied. Additional research is required to identify the specific interaction surface that could exist between these proteins. 

We previously observed that the interaction between BRCA1 tBRCT and RICTOR was phosphorylation independent and was not disrupted by phosphatase treatment [19]. Both the Y2H and co-IP experiments in this study were performed without the exogenous induction of DNA damage, but interactions with the mTORC2 subunits were still observed. While normal replicative stress can produce low-levels of DDR pathway activation, it is probable that these interactions are independent of phosphorylation status. It is important to note that while tBRCT domains were initially characterized as phospho-peptide binding motifs, many BRCA1 tBRCT interactions we discovered by Y2H were not disrupted by phosphatase treatment and presumably phosphorylation-independent [19]. Indeed, the phospho-peptide binding region of tBRCT domains occupies only a small portion of the solvent accessible surface of this modular domain [42]. Thus, phosphorylation-independent tBRCT interactions likely contribute to the overall scaffolding function of this domain.

The transcriptional activation activity of BRCA1 is central to its role in the DDR and requires a functional tBRCT domain. We sought to investigate the possible role mTORC1/2 signaling could play in BRCA1 transcriptional activation. At the concentration and exposure times used in our Rapamycin treatment experiments, Rapamycin specifically inhibited mTORC1 activity but not mTORC2. BRCA1 transcriptional activation increased significantly upon Rapamycin treatment but not when pan-mTORC1/2 PP242 inhibitor was used, indicating that mTORC1 negatively regulates BRCA1 transcriptional activation only when mTORC2 is active. The activity of mTORC2 is negatively regulated by mTORC1 in a negative feedback loop. It will be important to determine the mechanisms associated with mTORC1/2 regulation of BRCA1 transcriptional activity and the impact on the DDR.

Our results showed that there was depletion of BRCA1 foci upon inhibition of mTORC1/2. Similar results have been published that reveal Akt signaling regulates the localization and transcriptional activation of BRCA1 [43]. Impaired BRCA1 localization to DNA damage-induced foci may be the result of impaired signaling that disrupts phospho-peptide binding of the tBRCT. Indeed, our results indicated PP242 treatment in combination with Cisplatin also inhibited γH2AX formation. This suggests a defect prior to BRCA1 recruitment to DNA damage foci potentially affecting upstream kinase signaling, such as ATM/ATR that specifically phosphorylates γH2AX. The interplay between mTORC1/2 signaling on the DDR and the sensitivity of breast cancer cell lines with impaired *BRCA1* status to mTORC1/2 inhibition also suggests the potential for tailored treatment based on the patient’s underlying genetics.

The results of this study implicate mTORC2 signaling in the regulation of BRCA1 functions in the DDR. Components of the mTORC2 complex have been found to individually interact with the BRCA1 tBRCT domain. Rapamycin, which fails to inhibit mTORC2 activity, increases BRCA1 transcriptional co-activation. We also found that mTORC2 activity protects cells from cell death induced by Cisplatin treatment. Additionally, mTOR inhibition prevents γH2AX-BRCA1 co-localization, thus impairing the DDR. More importantly, sensitivity to mTOR inhibitors is dependent on *BRCA1* status. The information gained in this study could also be applied to different cancers because the BRCA1-mTORC2 interaction is broadly translatable. BRCA1 expression regulates the response to therapies, and both BRCA1 levels and mTORC2 activity are variable in tumors. The incorporation of metrics such as status of BRCA1, RICTOR, and activation of mTORC2 could guide patient selection for personalized therapies that may significantly increase the survival of breast cancer patients.

## 4. Materials and Methods 

### 4.1. Cell Culture and Treatment

MCF-10A cells were cultured in DMEM/F12 medium (Invitrogen, Carlsbad, CA, USA), containing 5% horse serum (Invitrogen, No. 16050-122), 20 ng/mL EGF (Sigma, St. Louis, MO, USA), 0.5 μg/mL Hydrocortisone (Sigma), 100 ng/mL Cholera toxin (Sigma), 10 μg/mL insulin (Sigma), and 1% penicillin-streptomycin (Invitrogen) at 37 °C in 5% humidified CO2 incubators. MDA-MB-231 cells were cultured in RPMI medium (Invitrogen), containing 10% FBS and 1% penicillin-streptomycin. MCF-7 and 293FT cells were cultured in DMEM medium (Invitrogen), containing 10% FBS and 1% penicillin-streptomycin. HCC1937 cells with pcDNA3-empty and pcDNA3-BRCA1 expression were a kind gift from the laboratory of Simon N. Powell. Transfection of 293FT cells to generate lentiviral particles was performed using the calcium phosphate method and the ViraPower system (Invitrogen). Target cells were transduced with virus, and 48 hours later, selected on puromycin (1 μg/mL) for 5–7 days.

### 4.2. Plasmids, Primers, and shRNAs

Lentiviral shRNA constructs in pLK0.1 targeting BRCA1 as well as nontargeting scrambled control, were purchased from Open Biosystems (Open Biosystems, Huntsville, AL, USA). The Gateway cloning system (Invitrogen) was used for the yeast two-hybrid screen. The amino acids 1650–1863 encompassing the two BRCA1 BRCT domains were used to clone constructs for both the yeast-two hybrid and co-overexpression experiments. The BRCA1 tandem BRCT domain baits (WT and M1775R) were cloned into the pDEST32 vector to generate GAL4 DNA binding domain fusions. The bait proteins (PRR5, SIN1, and RICTOR) were cloned into the pDEST22 vector to generate GAL4 activation domain fusions. The Myc-tagged vectors for the co-overexpression experiments were purchased from Origene (Origene, Rockville, MD, USA).

### 4.3. Yeast Two-Hybrid Screen

The Gateway-based ProQuest Two-Hybrid system (Invitrogen) was used to design and develop the yeast-two hybrid screen. MaV203 yeast cells were used for transformation of the corresponding GAL4 DNA binding domain and activation domain DNA constructs. After transformation, the cells were plated on dropout minimal media lacking the amino acids leucine and tryptophan to select clones containing both the bait and prey vectors. Once acceptable growth was shown on the double minus plates, two colonies were chosen from each plate to serve as “master colonies” for the further selective media plates. Each master colony was grown in selective -leu, -trp liquid medium to saturation. Prior to plating, each liquid culture was serially diluted 10-fold in phosphate buffered saline to test limiting numbers of cells in each singular sample. The yeast plates were also made with increasing concentrations of 3-amino-1, 2-triazole (3AT), an inhibitor of the HIS3 gene.

### 4.4. Transcriptional Activation Assays

The BRCA1 transcriptional activation assays were performed using a TA luciferase reporter assay as previously described [38]. The BRCA1 construct utilized in this assay spanned from amino acids 1315–1863 of the protein. BRCA1 constructs were co-transfected in 293FT cells with the pG5Luc plasmid, encoding a Luciferase reporter gene driven by GAL4 binding sites, and the phGR-TK plasmid, which constitutively expresses the internal control Renilla luciferase. Cells were either treated with 10 nM Rapamycin, 1 μM PP242, or DMSO control 24 h after transfection. Transcriptional activity was assayed with the Dual-Luciferase Reporter Assay System (Promega, Madison, WI, USA) 24 h after treatment. Each experiment measuring transcription activity was calculated by using a ratio of Firefly luciferase units to Renilla luciferase units. The average of three ratios from three experiments (*n* = 3) was quantified and plotted onto bar graphs. A student’s t-test was used to measure statistical significance.

### 4.5. Immunofluorescence

Cells were grown on coverslips and treated with 25 μM Cisplatin, 1 μM PP242, or 10 nM Rapamycin for 24 hours or a control and fixed in 4% paraformaldehyde for 15 min. Coverslips were washed in 0.5% Triton X-100/PBS for 1 min. Coverslips were then incubated with primary antibody in 3% BSA for 1 h. After the coverslips were washed three times with PBS, anti-rabbit AlexaFluor-488 secondary antibody and anti-mouse AlexaFluor-647 secondary antibody were added for 1 h. Coverslips were washed three times with PBS and mounted with DAPI counterstain mounting solution onto microscope slides. Foci from ≥ 300 cells were quantified and plotted onto bar graphs. A student’s t-test was used to determine statistical significance.

### 4.6. Western Blotting and Antibodies

Whole cell lysates were prepared using NETN lysis buffer (10 mM HEPES pH 7.4, 10 mM KCl, 0.05% NP-40). The lysis buffer contained phosphatase inhibitors (50 mM NaF, 10 mM β-glycerophosphate, 0.1 mM NaVO_4_) and a protease inhibitor cocktail (Sigma). Samples were mixed 1:4 with 5X Laemmli buffer and incubated at 95 °C for 5 min. Approximately 50–150 μg of protein was prepared for loading. The membrane was blocked in 5% non-fat milk and incubated with the primary antibody. BRCA1 (Ab-1) (OP92–100UG) antibody was purchased from Calbiochem (Calbiochem, San Diego, CA, USA). CBP (07–482) and RICTOR (05–1471) were purchased from Millipore (EMD Millipore, Burlington, MA, USA). phospho(Ser473)-AKT(#4691), phospho(Thr412/Thr398)-p70S6K I and II (#9204), AKT (#4060), total p-70S6K (#9202) were purchased from Cell Signaling Technology (Cell Signaling Technology, Danvers, MA, USA). MYC (MA1-21316) antibody was purchased from Thermo Scientific (Thermo Scientific, Waltham, MA, USA). HSC70 (sc-7298), β-actin (sc-47778), and α-tubulin (sc-53030) antibodies were purchased from Santa Cruz Biotechnology (Santa Cruz Biotechnology, Dallas, TX, USA). γH2AX (NB100-384) antibody was purchased from Novus Biologicals (Novus Biologicals, Littleton, CO, USA). Protein expression was visualized with ECL (Thermo Scientific) or near-IR secondary antibodies (LI-COR Biosciences, Lincoln, NE, USA) on the Odyssey Fc Imaging System (LI-COR Biosciences).

### 4.7. Cell Viability Assays

10,000 cells were plated into each of the wells of a black 96-well plate. Treatments were given 24 h after plating. After 72 h from plating, the Cell Titer Glo Luminescence Cell Viability Assay (Promega, Madison, WI, USA) was used to determine the cell viability of each of the experiments. The results of the plates were interpreted by using a 96-well plate reader. The averages of the intensity units were calculated and plotted onto a graph corresponding to the concentration of drug given using GraphPad Prism. For the EC50 studies, the averaged intensity units were normalized to percentages and plotted onto a graph with the corresponding concentration of drug given using GraphPad Prism.

## Abbreviations

BRCA1	Breast Cancer 1
DDR	DNA Damage Response
tBRCT	Tandem BRCA1 C-terminal domain
mTOR	Mammalian target of Rapamycin
RICTOR	Rapamycin-insensitive companion of mTOR
PRR5	Proline-rich protein 5
SIN1	Stress-activated map kinase-interacting protein 1
SEER	Surveillance, Epidemiology, and End Results
pSXXF	Phosphorylated Serine-X-X-Phenylalanine motif
TAP-MS	Tandem affinity purification coupled to mass spectrometry
Y2H	Yeast two-hybrid
WT	Wild type
DBD	DNA binding domain
AD	Activation domain
UAS	Upstream activation signal
